# Identification of environmental stressors and validation of light preference as a measure of anxiety in larval zebrafish

**DOI:** 10.1186/s12868-016-0298-z

**Published:** 2016-09-15

**Authors:** Yiming Bai, Harrison Liu, Bo Huang, Mahendra Wagle, Su Guo

**Affiliations:** 1 State Key Laboratory of Genetic Engineering, Department of Genetics, School of Life Sciences, Fudan University, Shanghai, China; 2 Department of Bioengineering and Therapeutic Sciences, Programs in Human Genetics and Biological Sciences, Wheeler Center for the Neurobiology of Addiction, University of California, San Francisco, CA USA; 3 Graduate Program in Bioengineering, University of California, San Francisco, CA USA; 4 Department of Pharmaceutical Chemistry, University of California, San Francisco, CA USA

**Keywords:** Zebrafish, Larvae, Light–dark preference, Anxiety, Stressor, Behavior, Cortisol

## Abstract

**Background:**

Larval zebrafish, with a simple and transparent vertebrate brain composed of ~100 K neurons, is well suited for deciphering entire neural circuit activity underlying behavior. Moreover, their small body size (~4–5 mm in length) is compatible with 96-well plates, making larval zebrafish amenable to high content screening. Despite these attractive features, there is a scarcity of behavioral characterizations in larval zebrafish compared to other model organisms as well as adult zebrafish.

**Results:**

In this study, we have characterized the physiological and behavioral responses of larval zebrafish to several easily amenable stimuli, including heat, cold, UV, mechanical disturbance (MD), and social isolation (SI). These stimuli are selected based on their perceived aversive nature to larval zebrafish. Using a light/dark choice paradigm, in which larval zebrafish display an innate dark avoidance behavior (i.e. scotophobia), we find that heat, cold and UV stimuli significantly enhance their dark avoidance with heat having the most striking effect, whereas MD and SI have little influence on the behavior. Surprisingly, using the cortisol assay, a physiological measure of stress, we uncover that all stimuli but heat and SI significantly increase the whole body cortisol levels.

**Conclusion:**

These results identify a series of stressors that can be easily administered to larval zebrafish. Those stimuli that elicit differential responses at behavioral and physiological levels warrant further studies at circuit levels to understand the underlying mechanisms. The findings that various stressors enhance while anxiolytics attenuate dark avoidance further reinforce that the light/dark preference behavior in larval zebrafish is fear/anxiety-associated.

## Background

The word “stress” refers to external factors that disrupt homeostasis. It was popularized by Hans Seyle through his books “The Stress of Life” and “Stress without Distress” [1, 2]. At present, the word “stressor” is often used to refer to the stimulus (either external or internal) that perturbs homeostasis and thus exerts an undesirable effect on physiology or behavior, whereas “stress” is more broadly used to describe either the stimulus or the state of being in an undesirable situation. Both acute and chronic stressors can lead to changes in behavior and physiology. The behaviors that are associated with stressors are often referred to as anxiety-related, manifesting as avoidance or aversion. Several classical behavioral assays, including the elevated plus-maze, the light/dark choice, and the open-field tests, have been used to measure anxiety-like behaviors in rodents, the animal models most widely used for stress research [3]. Zebrafish, a vertebrate genetic model developed in more recent years [4–9], also display a light/dark preference behavior: Adult zebrafish display scototaxis (or light avoidance), which is shown to be anxiety-related [10–12]. At the physiological level, the effect of stressors is commonly assessed through measuring the level of cortisol (or corticosterone in rodents), the release of which is under control of the hypothalamo-pituitary-adrenocortical (HPA) stress axis [13].

Studies of the effect of stress on behavior and physiology during the last century, conducted at cellular, molecular, physiological, and systems levels, have led to important knowledge on the brain and neuroendocrine structures involved (e.g. hypothalamus, amygdala, hippocampus, prefrontal cortex, locus coeruleus, raphe nucleus, pituitary gland, and adrenal gland) and associated neurochemical substrates (e.g. corticotropin-releasing factor-CRF, adrenocorticotropic hormone-ACTH, cortisol, noradrenaline serotonin, and neuropeptide Y) [14]. These studies have also led to the realization of a strong connection between stress and illness, including depression and anxiety disorders, addiction, cardiovascular abnormalities and cancer: chronic and uncontrollable stressors turn the acute adaptive response to maladaptive in the long run. Despite these significant advances, several challenges remain: What are the cell types that respond to various stressors and activate the stress axis? Once the stress axis is activated, how does it help coping with acute stressors, and how can it become maladaptive in the presence of chronic and uncontrollable stressors? What accounts for individual differences in stress susceptibility versus resilience?

Larval zebrafish represent an attractive model system to address these questions. The larval stage of zebrafish lasts from ~5 days post fertilization (dpf) to several weeks of age. During this period, they remain small (~several mms in length) and available in large quantities, thus making them suitable for high content screening. Moreover, they possess a simple and transparent brain with typical vertebrate anatomy and composed of ~100 K neurons. This feature makes them uniquely suited for whole brain neural activity imaging at cellular resolution [15–17], a technique that will greatly facilitate systems level understanding of neural circuitry underlying function. Starting at 5 dpf, larval zebrafish become free-living, hunt for food and escape from predators, suggesting that simple but functional circuitries for processing reward and aversion are already in place. However, compared to adult zebrafish and other model systems, we know relatively little about the extent of behavioral repertoire in larval zebrafish. It is presently unknown the various types of stressors that larval zebrafish may respond to at behavioral and physiological levels.

In this study, we selected five easily amenable stimuli of perceived aversive nature and characterized their effect on larval zebrafish at both behavioral and physiological levels. These stimuli included heat, cold, UV, mechanical disturbance (MD), and social isolation (SI). Excessive heat and cold outside the preferred temperature range of an organism is known to be stressful. UV is a potential stressor, as it increases oxidative stress in both embryonic and mature zebrafish [18]. MD in the form of restraining or chasing and SI have been shown to elicit stress responses in adult zebrafish [19, 20]. Upon administration of these stimuli, the behavioral responses of larval zebrafish were assessed using the light/dark choice assay, and their physiological responses were measured using the cortisol assay. Our results uncover an intriguing phenomenon that distinct stressors may preferentially elicit behavioral or physiological responses. Our data also reinforce that the light/dark choice assay is a sensitive measurement of anxiety-related behavior in larval zebrafish.

## Methods

### Animals and housing

Larval zebrafish (*D. rerio*) used for the experiments were from the AB strain bred in our facility at the University of California, San Francisco, CA and treated in accordance with IACUC regulations. AB wild type fish were crossed and kept from disturbance in the fish room. Embryos were collected in the late morning next day (age was set as post fertilization day 0, 0 dpf, days post fertilization) and then sorted into separate 100 mm Petri dishes filled with adequate egg water at no more than 40 embryos per dish. These embryos were raised at 28 °C from day 0 to day 2. At 3 dpf, the dishes were taken out of the incubator and exposed to the normal circadian cycle. The light intensity at the dish during day period was 350 lx. On the day before testing (5 dpf), 4 larvae were gently pipetted out of the 100 mm Petri dish and transferred to a 35 mm Petri dish filled with 7 ml 28 °C egg water (0.12 g of CaSO4, 0.2 g of Instant Ocean Salts from Aquatic Eco-systems, 30 μl of methylene blue in 1 L of H2O). Six 35 mm dishes with total 24 larvae was defined as a group, and for one round of experiments, 4 groups were used. In each group, 2 dishes were used for the behavioral test, and the rest 4 dishes were for the cortisol assay. All the behavioral tests and the cortisol collection were conducted at 6 dpf.

### Acute stressors

Larval fish were brought to the behavior room and allowed to habituate to the environment for 10 min. After the habituation, the larval fish were treated with various acute stressors for 5 min.

#### Control

For the control group, no stressors were applied to the fish. 10 ml 28 °C egg water was added with a transfer pipette to keep 17 ml 28 °C egg water in each dish.

#### Heat stimulus (Heat)

10 ml 50 °C egg water was added to each dish containing 7 ml 28 °C egg water with a transfer pipette to form a 17 ml 33 °C environment for larval fish. The treatment lasted for 5 min. Temperature of water was monitored, over the period of 5 min temperature gradually drops down from 33 °C and reaches to 30 °C by the end of 5 min.

#### Cold stimulus (Cold)

10 ml 4 °C egg water was added to each dish containing 7 ml 28 °C egg water with a transfer pipette to form a 17 ml 18 °C environment for larval fish. The treatment lasted for 5 min. Over the period of 5 min temperature rises from 18 °C and reaches to 23 °C by the end of 5 min.

#### UV light stimulus (UV)

6 dishes were placed on a white board under a UV light (SPECTROLINE MODEL ENF-280C). Then the larvae were treated with UV light (365 nm, 1.9–2.0 mW) for 5 min. Before the treatment, 10 ml 28 °C egg water was added with a transfer pipette to keep 17 ml 28 °C egg water in each dish.

#### Mechanical disturbance (MD)

10 ml 28 °C egg water was added with a transfer pipette to keep 17 ml 28 °C egg water in each dish. One mini stir-bar was put into each dish and then water was stirred on a stirrer (VWR-Hotplate/Stirrer or CORNING STIRRER/Hotplate) for 200 rpm, 5 min.

#### Social isolation (SI)

4 larvae from one 35 mm Petri dish were sorted into 4 individual petri-dishes (filled with 17 ml egg water). Blue, opaque tapes surrounding the side wall of each dish were used to ensure that an individual larva would not have any visual access to other conspecifics. The treatment lasted for 5 min.

### Behavioral test

#### Apparatus

A light box (STRATAGENE) was laid on a table with incandescent light bulbs placed on each side of the light box, illuminating the compartment with diffuse light. Black and white stripes, 5 cm wide and made from infrared-transmitting acrylic (ACRYLITE IR acrylic 11460), were then affixed to the top of the light box. The light intensity of light side was 2000 lx, and dark side 50 lx.

Each light/dark test apparatus consisted of four individual square tanks (L4 cm × W4 cm × H1.5 cm for each). Each tank was divided into two compartments of equal size, one white and one black. The sides of the compartments were surrounded by opaque white and black tape respectively in order to eliminate the transparency of the walls. We found larvae near the compartment walls to be difficult to track and thus added acrylic inserts as spacers. The inserts were made from infrared transmitting acrylic and were thus invisible in our videos. All acrylic components were designed in CorelDraw and fabricated using a laser cutter (Universal Laser Systems VersaLaser 3.50).

Each tank was filled with 10 ml 28 °C egg water (water depth: ~5 mm). It should be ensured that there is no air bubble in the tanks, and no water drops on the internal sides of the tanks. The water was changed in the tanks between each round of recording.

#### Light/dark choice assay

In the Light/dark choice assay, larval zebrafish were previously found to prefer light (or avoid dark) [12], and this behavior is thought to be anxiety-related. Increased percentage of time spent on the light side in larval zebrafish is thus believed to reflect increased anxiety. After 5-min stressor treatment, larval fish were transferred from the dish to the center of each tank with a plastic pipette, with minimal chasing.

#### Pharmacological treatment

Larvae were raised as described above. On the day of behavioral analysis (6 dpf), larvae were kept in egg water containing no drug (control), 100 μM chlordiazepoxide, or 75 μM Buspirone for 1 h. The drug concentration and length of treatment was determined by pilot experiments in which larvae were treated with various concentrations and the effect was monitored based on survival and locomotor activity.

#### Video tracking and data analysis

During behavioral testing, two apparatuses (totally 8 square tanks) were recorded simultaneously. One infrared camera (Panasonic CCTV camera Model No. WV-BP334) was set above each apparatus. A 8-min recording was made through the camera positioned above the tanks. The videos were recorded by Noldus MPEG Recorder 2.1 as digital video files. At the end of the 8-min recording, larval zebrafish were gathered in a dish to be euthanized with ice.

The position of the zebrafish larvae was traced using Ethovision XT 5.0 to analyze the digital video files. The output parameters included duration in the light zone or the dark zone, and swim velocity. For the light/dark choice assay, percentage of time spent in light/dark side and choice index were calculated over the 8-min recording period. Up to 10–15 % of samples were excluded as software failed to trace larvae due to high degree freezing and/or movement close to the walls of the chambers. Statistical analyses and graph generation were performed using the Graphpad Prism 5.0. Choice indices are presented as scattered plots showing individual sample values and error bars representing mean and 95 % CI. ANOVA followed by the Dunnett’s post hoc test was used to compare data for each stressor treatment with the control (no stressor treatment). Student t-tests (two tailed, unpaired) were performed for each treatment group compared to the control. For effect size, cohen’s d was calculated using d = 2t/√(df)

### Cortisol assay

#### Collection/freezing

Four larvae were collected at a time in ice-cold PBS, transferred to collection tubes, and placed on dry ice. Excess PBS was removed to ensure a final volume of 50 μl and stored t −70 °C till next step.

#### Homogenization and extraction

Samples were thawed on ice. 150 μl ice-cold PBS was added to each tube to ensure a total volume of 200 μl. Samples were homogenized with a handheld homogenizer (VWR Pellet Mixer #47747-370) for 30 s to 1 min. 20 μl of the tissue homogenate was kept in a separate tube, for total protein estimation. The remaining homogenate of 180 μl was added to 1400 μl of ethyl acetate for extraction. Tubes were centrifuged at 7000*g* for 15 min. The organic layer in each tube was transferred to a fresh tube and left to evaporation in the fume hood. Finally, the dried samples were dissolved in 180 μl of EIA buffer from the cortisol EIA kit.

#### Cortisol assay

The cortisol EIA kit (Cayman CHEMICAL) was used to conduct the cortisol assay. The experiment procedure followed the cortisol EIA kit protocol.

#### Protein assay

The BCA Protein Assay Kit (Pierce^®^ BCA Protein Assay Kit, Thermo SIENTIFIC) was used to conduct the protein assay. The experiment procedure followed the Pierce^®^ BCA Protein Assay Kit protocol.

#### Statistical analyses

Student t tests (two tailed, unpaired) were performed for each group compared to controls for the cortisol/protein assay.

## Results

### Rationale and design

Larval zebrafish display an innate preference for light (or avoidance of dark), and this choice is opposite to that of adult zebrafish [12]. The dark avoidance can be significantly alleviated by anxiolytic compounds [21, 22], suggesting that the behavior is anxiety-related. If the behavior was indeed anxiety-related, one would expect that stressors would have a modifying effect on the behavior that would be opposite to that of anxiolytics. This has, however, not been rigorously tested.

**Fig. 1:**
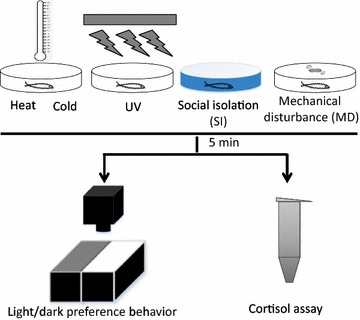
Schematics outlining the experimental design. The larval zebrafish were treated with various acute stressors (Heat stimulus, cold stimulus, UV light, social isolation and mechanical disturbance) for 5 min. After 5-min stressor treatment, larval zebrafish were transferred from the dish to the light/dark test apparatus for light/dark preference behavioral assay and recorded for 8 min, or collected with ice-cold phosphate buffered saline (PBS) and transferred to collection tubes on dry ice for cortisol assay

Here we chose five different stimuli based on their perceived aversive nature. These potential stressors are heat, cold, UV, mechanical disturbance (MD), and social isolation (SI). We have devised a number of methodologies to deliver these stimuli in a precise and quantifiable manner. All the exposure durations were kept identical. The larvae were exposed to acute stressors for 5 min as longer exposure was found to affect locomotor activity and freezing impacting the light/dark choice behavior. The behavioral responses of larval zebrafish to these stressors were measured using the light/dark choice assay, and their physiological responses were assessed using the whole body cortisol test (Fig. 1).

**Fig. 2:**
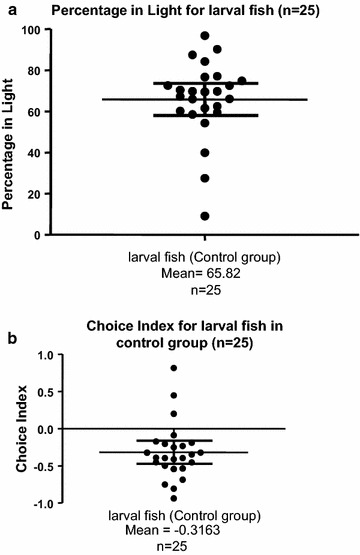
Performance of unstressed control larval zebrafish in the light/dark choice assay. **a** Percentage of total time spent in the light compartment for unstressed control larval zebrafish. **b** Choice Index for unstressed control larval zebrafish. Data are presented as scatter plots showing individual sample values and *error bars* representing mean and 95 % CI. Choice index was defined as: (Duration in dark − Duration in light)/(Duration in dark + Duration in light). One-sample t test compared to the theoretical mean of 0.5000 for percentage of total time spent in the light compartment, and the theoretical mean of 0.0 for choice index. Total n = 25

Student T tests were performed for analysis of effects of various stressors on percentage of time in light zone or dark zone, and also choice index (CI) in larval zebrafish. Choice index was defined as (Duration in dark − Duration in light)/(Duration in dark + Duration in light). Therefore, CI = 0 meant no light/dark preference, CI = −1 meant that the larva had spent all the time swimming in the light zone (the strongest light preference), whereas CI = 1 indicated that the larva had spent all the time swimming in the dark zone (the strongest dark preference). At the start, we used one-sample t test to assess the Light/Dark preference of larval fish in the control group (n = 25) and found that the control group showed significant light preference (an average of 65.8 % of time spent in light zone, *t*_(24)_ = 4.202, *p* = 0.0003, *d* = *1.715* compared to the theoretical mean of 50.0 %) (Fig. 2a) and choice for light zone (an average of −0.3163 of choice index, *t*_(24)_ = 4.202, *p* = 0.0003, *d* = *1.715* compared to the theoretical mean of 0.0) (Fig. 2b).

### Heat, cold, and UV stimuli significantly increase the dark avoidance behavior in the light/dark choice assay

**Fig. 3:**
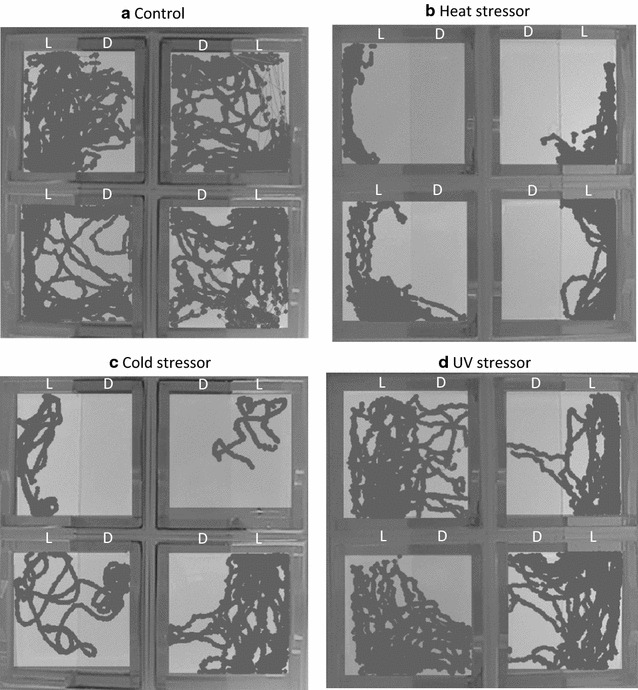
Images of movement traces of larval zebrafish exposed to heat, cold and UV stressors. **a** Representative movement traces of the control group. **b** Representative movement traces of the larval zebrafish after the treatment with heat stress. **c** Representative movement traces of the larval zebrafish after the treatment with cold stress. **d** Representative movement traces of the larval zebrafish after the treatment for UV light. *L* light compartment, *D* dark compartment

**Fig. 4:**
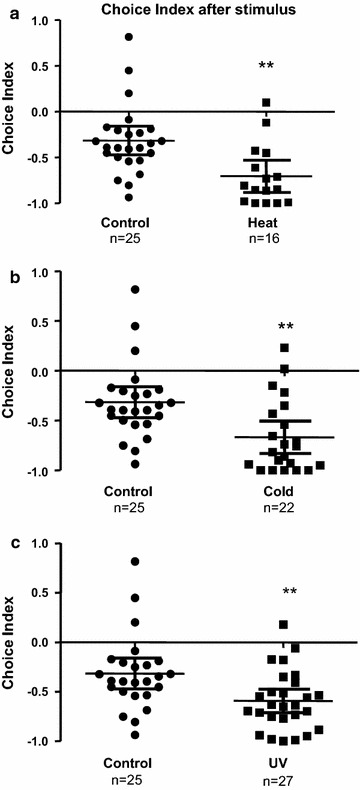
Computation of choice index reveals significantly increased dark avoidance in larval zebrafish exposed to heat, cold and UV stressors. **a** Choice index for the larval zebrafish after the treatment for heat stress. **b** Choice index for the larval zebrafish after the treatment for cold stress. **c** Choice index for the larval zebrafish after the treatment for UV light. Data are presented as scatter plots showing individual sample values and error bars representing mean and 95 % CI. **p < 0.01; student t tests compared to control (unstressed) group

We observed a significant effect of treatment for heat stress, cold stress and UV light on light zone preference according to the moving tract of larval zebrafish and statistical analyses. From the movement traces, we observed that, compared to the control group (Fig. 3a), larval fish spent significantly more time swimming in the light zone of the tank after the treatment for heat stress (Fig. 3b), cold stress (Fig. 3c) and UV light (Fig. 3d). Student T tests revealed that heat stress [*t*_(39)_ = 3.391, *p* = 0.0016, *d* = 1.086], cold stress [*t*_(45)_ = 3.240, *p* = 0.0023, *d* = 1.019] and UV light [*t*_(50)_ = 2.940, *p* = 0.0050, *d* = 0.831] all significantly increased the percentage of time spent in the light zone of the tank, decreased the percentage of time spent in the dark zone, and increased the absolute value of CI (Fig. 4a–c). The results indicate that after treatment with heat stress, cold stress or UV light, larval fish show significantly stronger dark avoidance relative to control.

### Social isolation and mechanical disturbance do not significantly alter the light/dark preference behavior

**Fig. 5:**
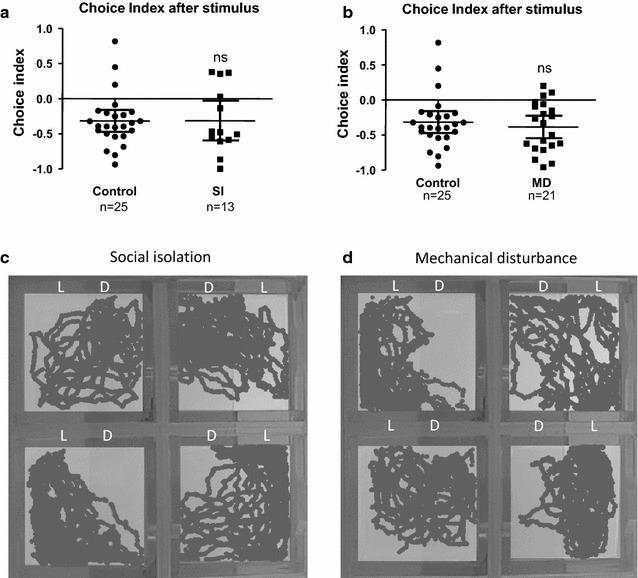
Social isolation and mechanical disturbance do not significantly alter the light/dark preference behavior. **a** Choice index for the larval zebrafish after the treatment for social isolation (SI). **b** Choice index for the larval zebrafish after the treatment for mechanical disturbance (MD). **c** Representative movement traces of larval zebrafish after the treatment with social isolation (SI). **d** Representative movement traces of larval zebrafish after the treatment with mechanical disturbance (MD). Data are presented as scatter plots showing individual sample values and error bars representing mean and 95 % CI. *ns* no significance; student t tests compared to control (unstressed) group

In contrast to the treatment with heat stress, cold stress and UV light, social isolation (SI) [*t*_(36)_ = 0.02, *p* = 0.9842*, d* = 0.006] and mechanical disturbance (MD) [*t*_(44)_ = 0.638, *p* = 0.5263, *d* = 0.192] showed no significant effect on the choice index (Fig. 5a, b). The movement traces also indicated that larval fish did not show a significant preference for the light zone after the treatment with these stimuli (Fig. 5c, d). These data suggest that social isolation and mechanical disturbance do not affect the light/dark preference behavior to a significant extent.

### The impact of the five stressors on locomotor behaviors

**Fig. 6:**
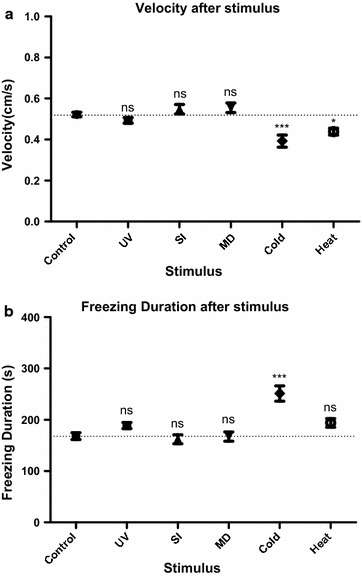
The effect of various stressors on locomotor behaviors. **a** Swim velocity measured as cm/s traveled. **b** Freezing duration. Data are presented as mean ± SEM; ***p < 0.001, *p < 0.05; post hoc Dunnett’s Multiple Comparison test after one way ANOVA compared to control (unstressed) group

Changes in the light/dark preference behavior upon exposure to various stressors could be due to alterations in either internal emotional states (e.g. anxiety-related) or sensorimotor capabilities. Sensory deficits are unlikely, since the stressors of heat, cold, and UV increased rather than abolished the light/dark preference behavior, suggesting intact visual abilities to guide preference. We determined the impact of the five stressors on locomotor behaviors, as changes in locomotor patterns might physically impede the capability for place preference. Additionally, such changes (e.g. an increase in freezing) might provide additional measures of anxiety-like states. The velocity of groups treated with heat stimulus and cold stimulus decreased significantly compared to the control group, while UV light, social isolation and mechanical disturbance showed no significant effect. As the behavioral chamber is small-sized, the extent of decrease in velocity would not prevent them from exploring the entire chamber (see examples of movement traces in Fig. 3), thus could not account for altered preference. Among all five stressors, the cold stress significantly increased the freezing duration of larval fish (Fig. 6). The locomotor behavioral analysis is consistent with the stressors affecting internal states rather than locomotor capabilities.

### The effect of the five stressors on the cortisol level exhibits discordance with their effects on the light/dark choice behavior

**Fig. 7:**
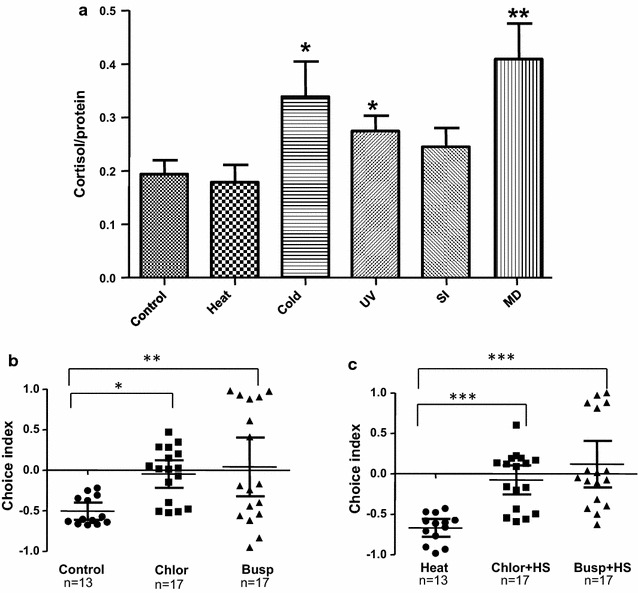
Physiological effects of various stressors and effect of anxiolytic drugs on non-stressed and stressed larvae (**a**) Relative cortisol level (absolute cortisol concentration/total protein concentration). Data are presented as mean ± SEM; **p < 0.01, *p < 0.05, *ns* no significance; post hoc Dunnett’s Multiple Comparison test after one way ANOVA compared to control (unstressed) group. Effect of chlordiazepoxide (Chlor) and Buspirone (Busp) on light/dark choice index of non-stressed (**b**) and heat stressed (HS) (**c**) larvae. Data are presented as scatter plots showing individual sample values and error bars representing mean and 95 % CI. *p < 0.05, **p < 0.01, ***p < 0.001; n = 13 for control and heat, n = 17 for all other groups, post hoc Dunnett’s Multiple Comparison test after one way ANOVA compared to control (no drug) group

Taken together, our data showed that significantly stronger dark avoidance could be evoked by treatment with heat stress, cold stress and UV light, but not with social isolation and mechanical disturbance. We next measured the relative cortisol levels (absolute cortisol concentration/total protein concentration), which is generally considered as a physiological indicator for anxiety. The relative cortisol levels of the larval fish treated with cold stress, UV light and mechanical disturbance increased significantly, but heat stress and social isolation exerted no significant effects on the relative cortisol levels (Student *T* test, individual group compare to control *p < 0.05, **p < 0.005) (Fig. 7a). Of particular note, while the treatment with heat stress induced the most robust dark avoidance, it did not significantly influence the relative cortisol level. By contrast, mechanical disturbance led to the highest rise of the relative cortisol level but did not bring about any change in the light/dark preference behavior. The other three stressors, cold, UV light and social isolation, elicited consistent responses in the cortisol assay and the light/dark preference test: Cold and UV increased both dark avoidance and cortisol, whereas social isolation affected neither.

### Link between anxiety and larval light/dark choice behavior

Although previous studies have shown that anxiolytics can decrease baseline dark avoidance behavior in larval zebrafish [21, 22], it is not known whether they have any effect on larval zebrafish subjected to stressful conditions, which might better resemble anxiety-like states. We thus carried out experiments to determine whether two commonly used anxiolytics used to treat human anxiety disorders, chlordiazepoxide and buspirone, would affect dark avoidance aggravated by the heat stress. In pilot experiment we determined the concentration of anxiolytics and length of treatment that did not have any effect on locomotor capability (data not shown). Larvae pretreated for 1 h with 100 μM chlordiazepoxide or 75 μM buspirone showed significantly reduced dark avoidance in non-stressed conditions [*F*(2, 44) = 5.350, *p* = 0.008] (Fig. 7b) (One way ANOVA and post hoc Dunnett’s Multiple Comparison test). Larvae pre-treated with anxiolytics were exposed to heat stress as described above. Dark avoidance in these heat stressed larvae was also found to be significantly reduced [*F*(2, 44) = 14.33, *p* < 0.0001] (Fig. 7c). Therefore, these data suggest that the light/dark preference behavior in larval zebrafish is indeed anxiety-associated.

## Discussion

**Fig. 8:**
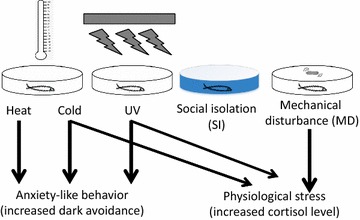
Schematic summary of the findings presented in this study. (1) While the treatment with heat stress induced robust dark avoidance, it did not significantly influence the relative cortisol level. (2) By contrast, mechanical disturbance led to the highest rise of the relative cortisol level but did not bring about any change in the light/dark preference behavior. (3) The other three stressors, cold, UV light and social isolation, elicited consistent responses in the cortisol assay and the light/dark preference behavior: Cold and UV increased both dark avoidance and cortisol, whereas social isolation affected neither

This study reports several important findings (Fig. 8): First, it identified stressors that elicited significant behavioral and/or physiological responses in larval zebrafish, an attractive vertebrate model system for behavioral genetics and systems neuroscience. Most of the stressors (e.g. heat, cold, or UV) that we have identified can be easily delivered to immobile animals during whole-brain imaging. To our knowledge, this represents the first comprehensive report on stressor-elicited behavioral and physiological changes in larval zebrafish.

Second, this study provides further validation that the light/dark choice behavior assay measures anxiety-like states in larval zebrafish. In recent years, several groups have reported that zebrafish exhibit innate preferences for differentially illuminated environments: While adult zebrafish clearly avoid light [11, 12], larval zebrafish have been found to display an opposite preference, that is, dark avoidance [12]. Light avoidance in adult zebrafish has good face validity (i.e. hiding, predator avoidance) and is oppositely modulated by stressors and anxiolytic compounds, hence demonstrating good construct and predictive validities [23]. In contrast, dark avoidance as a behavioral indicator of anxiety in larval zebrafish has been met with skepticism, despite that the behavior is shown modulated by widely known anxiolytics [21, 22]. Here, by demonstrating that multiple stressors with good face validity enhance dark avoidance in larval zebrafish, and moreover, such dark avoidance aggravated by the heat stress can be significantly alleviated by these anxiolytics, we provide key evidence that dark avoidance in larval zebrafish is indeed anxiety-related. It is ethologically conceivable that a dark environment, which may represent the shadow of a roving predator or signal lack of warmth, is undesirable to larval zebrafish.

Third, we discover an intriguing discordance between behavioral and physiological measures of stress. Cold stress and UV exposure increase both dark avoidance and cortisol level; in contrast, the heat stress only increases dark avoidance, and mechanical disturbance only increases cortisol level, with each being the most robust stimulus in their respective assays. One explanation for these discrepancies is that different stressors produce different temporal profiles in cortisol levels, a possibility our assays cannot formally exclude. However, the dissociation between the behavioral and physiological measures of stress that we observe is consistent with the idea that there exist a number of anxiety-related states each regulated by a distinct neuronal circuit and activated by different stressors.

Finally, acute social isolation affected neither light/dark preference nor cortisol levels in larval zebrafish. This observation is consistent with the finding that social preference develops with age in zebrafish [24]. It is possible that prolonged social isolation might exert an effect. Interestingly, chronic social isolation applied to adult zebrafish surprisingly decreases anxiety-like behaviors accompanied by a decrease in serotonin levels [25]. Together, how the presence or absence of conspecifics affect emotional behaviors warrant further exploration in the future.

## Conclusion

A series of stressors including heat, cold, UV, and mechanical disturbance, which can be easily administered to larval zebrafish are identified that increase dark avoidance behavior and/or whole body cortisol levels. This finding, together with the notion that anxiolytics attenuate dark avoidance further reinforce that the light/dark preference behavior in larval zebrafish is fear/anxiety-associated.

## Abbreviations

dpf: days post fertilization
MD: mechanical disturbance
SI: social isolation
HPA: hypothalamo-pituitary-adrenocortical
